# CD82 and Gangliosides Tune CD81 Membrane Behavior

**DOI:** 10.3390/ijms22168459

**Published:** 2021-08-06

**Authors:** Laurent Fernandez, Morgane Malrieu, Christine Bénistant, Patrice Dosset, Eric Rubinstein, Elena Odintsova, Fedor Berditchevski, Pierre-Emmanuel Milhiet

**Affiliations:** 1 Centre de Biologie Structurale (CBS), INSERM (National Institute of Health and Medical Research), CNRS (Centre National de la Recherche Scientifique), Université de Montpellier, 34090 Montpellier, France; laurentfernandez281288@gmail.com (L.F.); morgane.malrieu@gmail.com (M.M.); christine.benistant@cbs.cnrs.fr (C.B.); pat@cbs.cnrs.fr (P.D.); 2 INSERM (National Institute of Health and Medical Research), U602, 94807 Villejuif, France; eric.rubinstein@inserm.fr; 3 Institut André Lwoff, Université Paris 11, 94807 Villejuif, France; 4 Institute of Cancer and Genomic Sciences, University of Birmingham, Edgbaston, Birmingham B15 2TT, UK; e.odintsova@bham.ac.uk (E.O.); F.BERDITCHEVSKI@bham.ac.uk (F.B.); 5 Centre de Biologie Structurale, 29, rue de Navacelles, 34090 Montpellier, France

**Keywords:** tetraspanins, CD81, CD82, gangliosides, single-molecule tracking, microdomain, membrane diffusion, fluorescence microscopy

## Abstract

Tetraspanins are a family of transmembrane proteins that form a network of protein–protein interactions within the plasma membrane. Within this network, tetraspanin are thought to control the lateral segregation of their partners at the plasma membrane through mechanisms involving specific lipids. Here, we used a single molecule tracking approach to study the membrane behavior of tetraspanins in mammary epithelial cells and demonstrate that despite a common overall behavior, each tetraspanin (CD9, CD81 and CD82) has a specific signature in terms of dynamics. Furthermore, we demonstrated that tetraspanin dynamics on the cell surface are dependent on gangliosides. More specifically, we found that CD82 expression increases the dynamics of CD81 and alters its localization at the plasma membrane, this has no effect on the behavior of CD9. Our results provide new information on the ability of CD82 and gangliosides to differentially modulate the dynamics and organization of tetraspanins at the plasma membrane and highlight that its lipid and protein composition is involved in the dynamical architecture of the tetraspanin web. We predict that CD82 may act as a regulator of the lateral segregation of specific tetraspanins at the plasma membrane while gangliosides could play a crucial role in establishing tetraspanin-enriched areas.

## 1. Introduction

Tetraspanins are transmembrane proteins characterized by four membrane spanning hydrophobic domains, delineating two extracellular domains [1]. The large extracellular loop contains conserved residues, which contribute to the formation of a fold specific for tetraspanins [2]. Tetraspanins have been shown to modulate adhesion strengthening, cell migration, receptor signaling, cell–cell fusion [3] and trafficking of associated proteins [4]. Some tetraspanins, including CD82, have been associated with cancer progression [5,6,7]. This involvement in a wide spectrum of cellular processes can be explained by the ability of tetraspanins to form a network of protein–protein interactions within membranes, the so-called tetraspanin webs (or tetraspanin-enriched microdomains (TEM or TERM)) that rely on the homo- or hetero-multimerization of tetraspanins and stable interactions with other membrane proteins [8]. Importantly, using advanced fluorescence microscopy techniques, we and others have demonstrated that several tetraspanins (e.g., CD9, CD81 and CD151) are dynamic molecules that can diffuse within the plasma membrane [9,10]. Using Single Molecule Tracking (SMT), two main modes of motion were identified: Brownian and confined with a combination of these two modes corresponding to the transient confinement of proteins [11,12]. Specifically, we demonstrated that tetraspanins are often confined within specific tetraspanins-enriched areas (TEAs) and that these areas form stable platforms in permanent exchange with the rest of the membrane [13]. Moreover, the functional relevance of this dynamics has been highlighted in the context of viral infection [14,15] and cell migration [16,17].

The tetraspanin CD82 (also known as KAI-1) has been identified as a metastasis suppressor in prostate cancer [18]. Subsequent studies demonstrated that the expression of CD82 protein is frequently lost or downregulated during tumor progression in various epithelial cancers [7]. While the role of CD82 in the modulation of cancer-/metastasis-related pathways is not well understood, several studies have demonstrated that CD82 influences tumor cell migration and invasion by affecting integrin function or through association with EWI-2 and CD81 [19,20,21,22]. We also found that CD82 attenuates EGF-induced signaling [22] and regulates compartmentalization and dimerization of ErbB receptors [23]. Importantly, some of the CD82-dependent functions appear to be linked to gangliosides, glycosphingolipids found in the outer leaflet of the plasma membrane. Gangliosides have been proposed to form molecular assemblies containing tetraspanins and their partners (also referred to as glycosynapses [24]). In this regard, it has been demonstrated that various gangliosides modulate interactions of CD82 with its molecular partners [23]. Specifically, ganglioside depletion has a negative effect on the interaction between CD82 and EGFR, CD9, α_3_β_1_ integrin and CD151. In another study CD82 was shown to interact directly with the gangliosides GM2 and GM3, impairing cell migration by decreasing EGFR expression and phosphorylation and cMet activation [25]. Taken together, these observations support the idea that gangliosides could regulate, at least in part, the function of CD82-contaning complexes.

In this work, we examined how CD82 and gangliosides can affect the lateral organization of the tetraspanins CD9 and CD81 at the plasma membrane of non-malignant human mammary epithelial cell line (HB2 cells) using SMT, a technique pioneered by Schindler’s group in biological membranes using dye molecules [26], allowing the observation of the motion of individual particles. This technique has been applied to investigate the motion of lipids and proteins within membranes and largely used in the context of raft microdomains (see the review [27]). As mentioned above, we already implemented in our group such a technique to study tetraspanin lateral segregation based on the use of monovalent Fab fragments directly labelled with a chemical dye to prevent the potential steric hindrance sometimes observed with quantum dots [28]. We used here SMT, combined with an automatic classification of membrane behavior of single transmembrane proteins based on neural network [29], to compare the membrane behavior of CD82 with that of two other tetraspanins, CD9 and CD81. Our experiments revealed that despite overlapping molecular partnerships, all three tetraspanin proteins display significant differences in their behavior at the plasma membrane. Specifically, we demonstrate that while CD82 expression increases the dynamics of CD81 and modifies its localization at the plasma membrane, the behavior and cellular distribution of CD9 is not modified. Finally, our results suggest that gangliosides are not involved in CD82-dependent changes of CD81 dynamics but rather modulate the overall organization of tetraspanins into TEAs.

## 2. Results

### 2.1. Tetraspanins Display Different Dynamics at the Plasma Membrane of HB2 Cells

We have previously demonstrated that the tetraspanins CD9 and CD81, which are structurally related, are highly dynamic molecules at the plasma membrane of epithelial cells (PC3, HeLa, HuH7 and HepG2) [13,30,31]. Despite their high mobility, a significant number of proteins can be temporarily confined in TEAs. In this study, we wanted to extend these observations and investigate the behavior of CD82, a diverged member of the tetraspanin family. In these experiments we used HB2 cells [22], which constitutively express CD9 and CD81. We first performed SMT experiments on HB2/CD82 cells plated on coverslips and labelled with Fab fragments of antibody coupled to Atto647N and raised against the tetraspanin of interest. The advantages of Fab fragments are their small size and that they are monovalent but we cannot completely exclude that their binding could perturb the tetraspanin network. We are however very confident about our results since we used a very low concentration of Fab fragments (see Methods for details) in order to reach single-molecule imaging conditions and similar results about CD9 and CD81 partitioning were obtained in HeLa cells in which the membrane partnership is probably different from that of HB2 cells [15]. The recorded movies (see Supplemental Movies 1, 2 and 3 for representative examples and Figure S1) were then analyzed using our in-house software Patrack based on MSD (Mean Square displacement) analysis [29] (see details in the Materials and Methods section).

**Figure 1:**
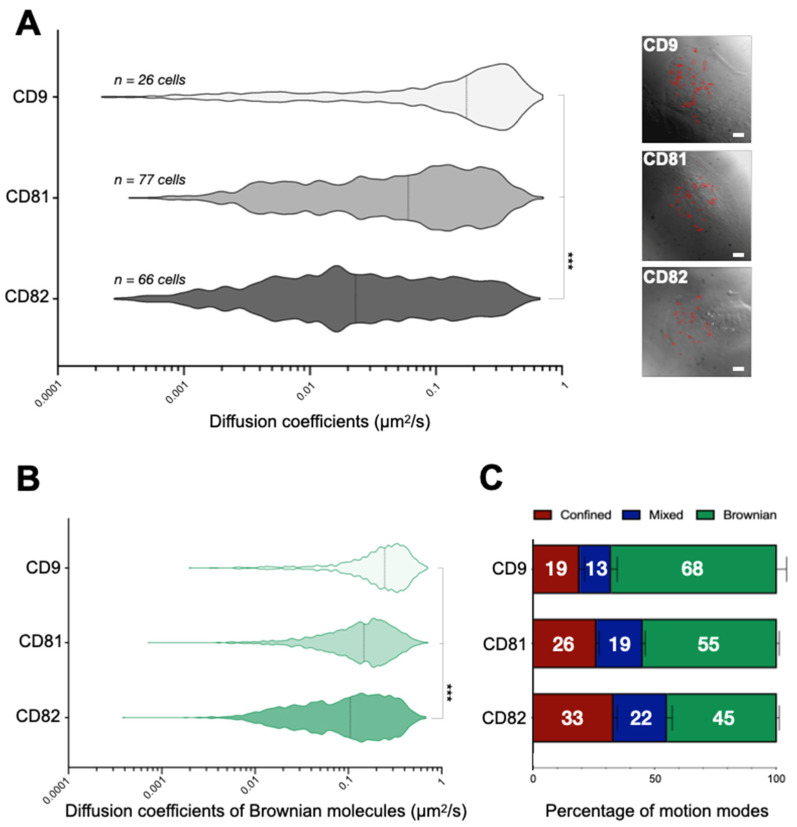
Membrane dynamics of tetraspanins CD82, CD81, and CD9 at the plasma membrane of HB2/CD82 cells. (**A**) Distribution of the apparent diffusion coefficients calculated for all individual tetraspanins molecules analyzed in HB2/CD82 cells. The violin plots were built with 1000 trajectories for each tetraspanin. The dotted lines indicate the median of the populations. The images on the right are examples of DIC images of cells taken after single molecule tracking and some analyzed trajectories are shown in red (scale bars, 5 µm). (**B**) Distribution of the apparent diffusion coefficients of Brownian tetraspanin molecules analyzed in HB2/CD82 cells. The dotted lines indicate the median of the populations. *** indicate that the difference between the populations is significant with a *p* value below 0.0001 as determined by the Mann–Whitney U test. (**C**) Histograms representing the percentage of tetraspanin molecules exhibiting Brownian, confined and mixed motion relative to the total number of trajectories. The error bars represent the standard deviation of at least three independent experiments.

Although all three tetraspanins display similar diffusion modes already described for CD9 [13], namely pure Brownian, confined, or a combination of Brownian and confined diffusion (referred to as mixed diffusion), we observed notable differences between the dynamics of the proteins at the plasma membrane of HB2/CD82 cells (Figure 1A,B). Specifically, we found that CD9 is more dynamic than CD81, which, in turn, is more dynamic than CD82 (Figure 1A,B). Indeed, the median diffusion coefficients of CD9, CD81 and CD82 are respectively 0.17, 0.06 and 0.03 μm^2^/s (median values of apparent diffusion coefficients are calculated from 1000 trajectories, Figure 1A). These differences are due in part to the fact that a higher proportion of CD9 molecules undergoes pure Brownian trajectories, the diffusion of which is faster than that of mixed and confined trajectories (68% as compared to 55% and 45% for CD81 and CD82). Furthermore, we also observed that the rate of diffusion of CD9 molecules exhibiting Brownian motion is faster than that of CD81 and CD82 molecules (median = 0.25 μm^2^/s vs. 0.15 μm^2^/s and 0.10 μm^2^/s, respectively). We also tracked CD82 proteins in HB2/Zeo and observed the same behavior with a median diffusion coefficient in the same range (0.02 µm^2^/s).

### 2.2. CD82 Specifically Increases CD81 Dynamics at the Plasma Membrane of HB2 Cells

**Figure 2:**
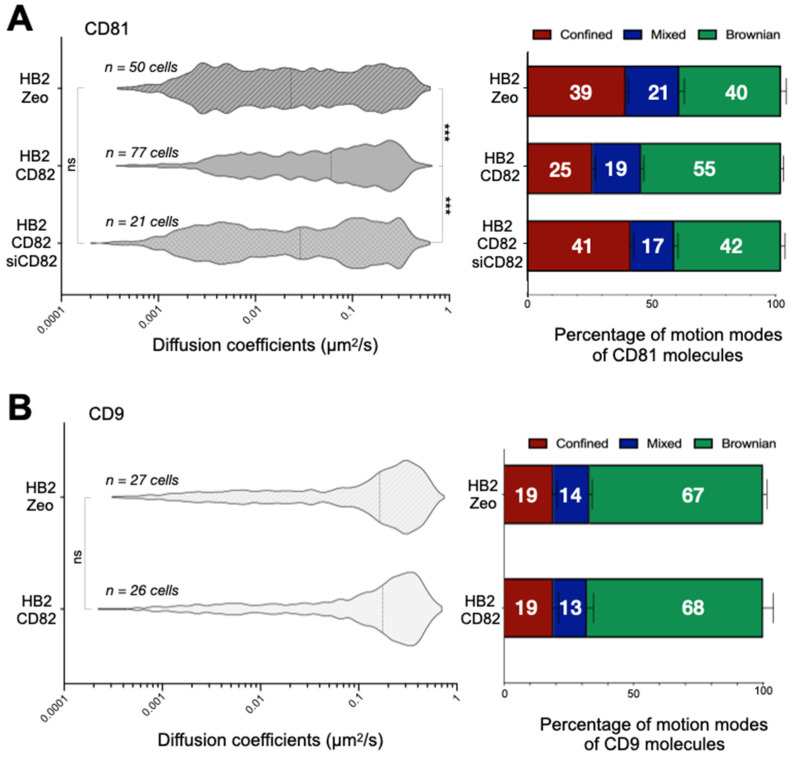
Left Panels: Distribution of the apparent diffusion coefficients calculated for all individual CD81 (**A**) or CD9 (**B**) molecules at the plasma membrane of HB2/CD82, HB2/zeo cells or HB2/CD82 transfected with siRNA targeting CD82. The violin plots were built with 1000 trajectories for each tetraspanin. The dotted lines indicate the median of the populations. *** indicate that the difference between the populations is significant with a *p* value below 0.0001 as determined by the Mann–Whitney U test («ns» for non-significant). Right Panels: Histograms representing the percentage of CD81 (**A**) or CD9 (**B**) molecules exhibiting Brownian, confined and mixed motion relative to the total number of trajectories. The error bars represent the standard deviation of at least three independent experiments.

The differential dynamics of CD9, CD81 and CD82 described above could be due to tetraspanin-specific differences in forming molecular partnerships with other proteins on the plasma membrane. Firstly, we investigated whether the dynamics of CD9 and CD81, well established CD82 partners, were affected by this tetraspanin. Specifically, we compared the behavior of CD81 and CD9 proteins in HB2/CD82 and HB2/Zeo cells (the expression level of CD82 in HB2/Zeo cells was approximately ten times lower than that seen in HB2/CD82 cells, Figure S2) [29]. These experiments demonstrated that CD82 expression had no impact on CD9 behavior but strongly modified the behavior of CD81. Specifically, the median diffusion coefficient of CD81 in HB2/Zeo cells was three times lower compared to HB2/CD82 cells (Figure 2A). This was partly due to a lower proportion of Brownian CD81 molecules in these cells on one hand (40% in HB2/zeo cells as compared to 55% in HB2/CD82) and a higher proportion of confined molecules on the other hand (39% in HB2/Zeo cells vs. 25% in HB2/CD82 cells). The specific effect of CD82 on CD81 dynamics was further supported in reverse experiments in which CD82 knockdown using RNAi resulted in a decrease of CD81 diffusion coefficient at the plasma membrane (Figure 2A) to a value close to a value comparable to that seen in HB2/Zeo cells. Interestingly, CD82 did not influence the diffusion coefficients of CD81 molecules displaying Brownian motion, but led to an increase of the diffusion coefficients of both mixed and confined CD81 molecules (Figure S3A). These results suggest that CD82 reduces the interaction of CD81 with membrane or juxtamembrane component(s), which may restrict its diffusion. Importantly, expression levels of CD9 and CD81 were comparable in HB2/Zeo and HB2/CD82 cells (Figure S2C).

In other experiments, we observed that the dynamics of the α_3_β_1_ integrin, which interacts indirectly with CD82 though another tetraspanin, CD151, was not affected by the expression of CD82 (Figure S2A).

### 2.3. CD82 Modulates the Localization of CD81 at the Plasma Membrane

**Figure 3:**
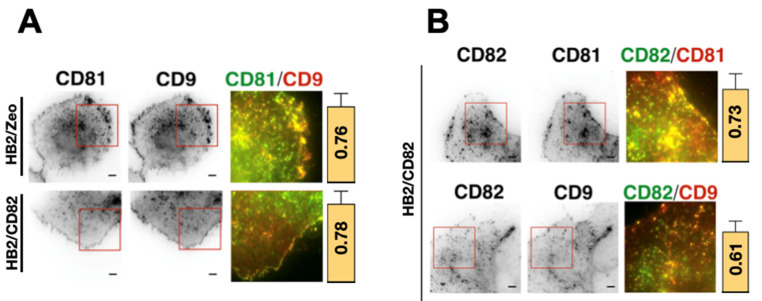
Ensemble labelling of CD9, CD81 and CD82 in HB2/Zeo and HB2/CD82 cells. (**A**) CD9-CD81 colocalization in both cell lines; (**B**) CD82-CD9 or CD81 in HB2/CD82 cells. The scale bar represents 5 μm. Images on the right part of both panels correspond to zooms delineated by the red boxes and represent the merge of the two channels. The yellow pixels represent the colocalization between tetraspanins. The histograms represent the calculated Pearson’s correlation coefficients between the two signals in HB2/Zeo and HB2/CD82 cells using the «colocalizer studio» plugin of the open-source community image processing software Icy (Release 1.9.4.0). Calculations were done on at least 10 cells and the error bars represent the standard deviation.

The increase of CD81 confinement in cells expressing high levels of CD82 strengthened the idea that CD82 could affect the partnership of tetraspanins within the tetraspanin web. To test this hypothesis, we examined co-localization of CD82, CD9 and CD81 at the plasma membrane of HB2/Zeo and HB2/CD82 cells using ensemble labeling (term used to describe a conventional labeling with saturation of the antigenic sites as compared to single molecule detection) combined with TIRF microscopy (Figure 3A). As previously reported for other cell lines [13,32], these tetraspanins were enriched in dot-like structures or larger patches at the basal membrane (referred to as TEAs). Many of these structures contained at least two of the 3 tetraspanins, albeit at different relative ratios. Quantification of the colocalization by Pearson correlation coefficient (PCC) analysis revealed that the expression of CD82 did not affect the high degree of colocalization of CD81 and CD9 (Figure 3A). Interestingly, CD82 expression led to an enrichment of CD9 and CD81 at the cell periphery in most of the imaged cells.

We also evaluated CD82-CD81 and CD82-CD9 colocalization in HB2/CD82 cells. As for CD9 and CD81, CD82 was found in dot-like structures and in larger patches at the cell membrane of HB2/CD82 cells (Figure 3B). The calculated PCC showed that both CD9 and CD81 are well co-localized with CD82 at the basal membrane of HB2/CD82 cells: the PCC was 73% for CD82 and CD81 and 61% for CD82 and CD9 (Figure 3B).

### 2.4. Effect of Gangliosides on the Dynamics and Organization of Tetraspanins

A functional link between CD9 and CD82 and gangliosides has been previously reported [33]. Notably, CD82 was shown to upregulate the expression of the gangliosides GM1, GD1a [23] and GM3 [24]. To extend this finding, we analyzed the lipid composition of HB2/Zeo and HB2/CD82 cells using a lipidomic approach (of note this approach does not allow quantification of GM1 due to technical limitations). Cellular levels of GD1a and GM3 were almost doubled up in CD82-expressing HB2 cells with only a slight increase in the amount of GM2. By contrast, there were no significant changes in the quantity of phosphatidylcholine (PC), phosphatidyl ethanolamine (PE), sphingomyelin (SM) and cholesterol in HB2/CD82 cells compared to HB2/zeo cells (Figure S4A). Thus, this first lipidomic approach on tetraspanins demonstrates that CD82 expression specifically affects the expression of a subset of gangliosides.

**Figure 4:**
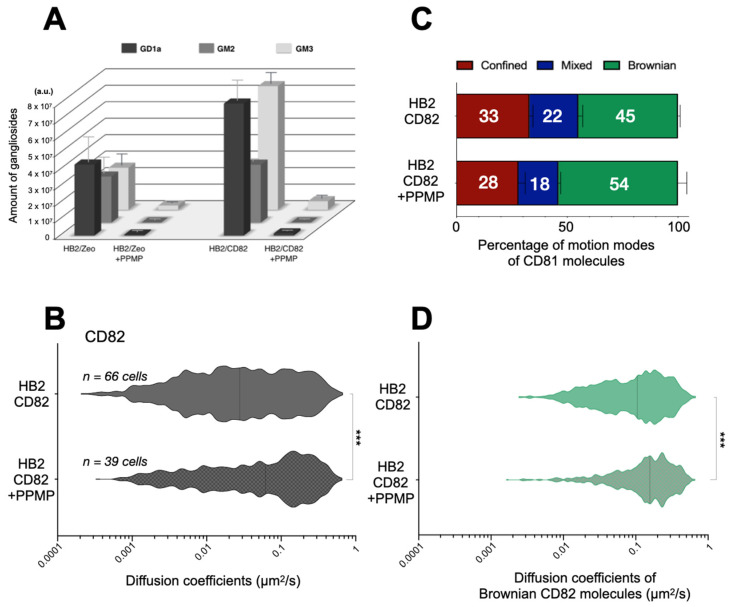
(**A**) Mass spectrometry analysis of ganglioside expression in HB2/Zeo or HB2/CD82 treated or not with PPMP. The analyses have been done in triplicate with samples containing 10^6^ cells. The error bars represent the SEM of three independent experiments. (**B**) Distribution of the apparent diffusion coefficients calculated for all the individual CD82 molecules analyzed in HB2/CD82 cells treated or not with PPMP. The violin plots were built with 1000 trajectories for each condition. (**C**) Histograms representing the percentage of CD82 molecules exhibiting Brownian, confined and mixed mode relative to the total number of trajectories in HB2/CD82 cells treated or not with PPMP. The error bars represent the standard deviation of at least three independent experiments. (**D**) Distribution of the apparent diffusion coefficients of Brownian CD82 molecules. *** indicate that the difference between the two populations is significant with a *p* value below 0.0001 as determined by the Mann–Whitney U test.

Ganglioside expression can be lowered by treating the cells with the ceramide analogue D-*threo*-1-phenyl-2-hexadecanoylamino-3-morpholino-1-propanol·HCl (PPMP). This molecule acts very early in the ganglioside synthesis pathway by inhibiting the glucosyl ceramide (GlcCer) synthase (GlcCer is the first precursor of gangliosides) [34]. To verify the specificity of this treatment in our experimental models, we analyzed the lipid composition of cells treated with this reagent. As expected, treatment with PPMP strongly decreased the cellular levels of all the three gangliosides GD1a, GM2 and GM3 (Figure 4A), and had no effect on the levels of cholesterol and PC (Figure S4A). Interestingly, treatment with PPMP also resulted in a slight decrease in the level of PE and an increase of SM. Importantly, expression of tetraspanins [35] and interaction between them (see immunoprecipitation in Figure S5) were preserved after PPMP treatment.

First, we studied the effect of gangliosides on CD82 dynamics in HB2/CD82 cells. The overall median diffusion coefficient of CD82 doubled from 0.03 μm^2^/s in HB2/CD82 to 0.06 μm^2^/s in PPMP-treated HB2/CD82 cells (Figure 4B). This was due in part to a higher fraction of CD82 molecules exhibiting Brownian motion upon PPMP treatment. Importantly, PPMP also impacted the diffusion coefficients of CD82 molecules displaying Brownian behavior (Figure 4C,D): the median diffusion coefficient of Brownian CD82 molecules increased from 0.10 μm^2^/s in HB2/CD82 cells to 0.16 μm^2^/s in PPMP-treated HB2/CD82 cells (Figure 4D). This increase was mainly due to the loss of a population of Brownian CD82 molecules diffusing slowly.

**Figure 5:**
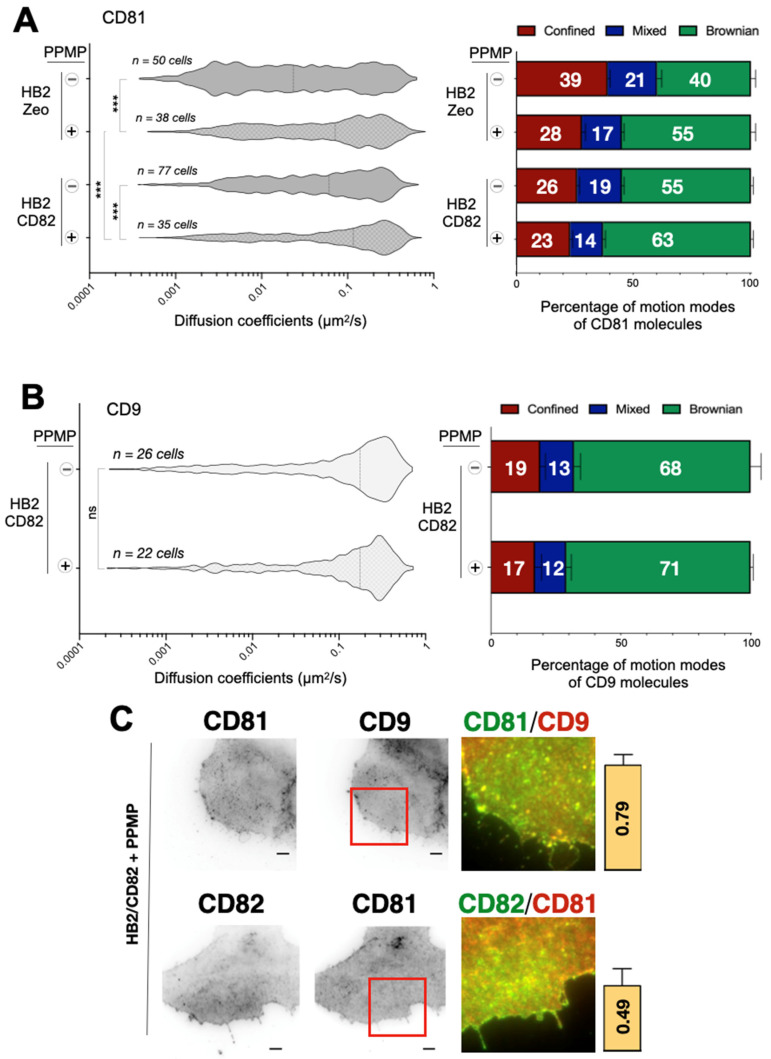
(**A**) Left: Distribution of the apparent diffusion coefficients calculated for all individual CD81 molecules analyzed in HB2/CD82 and HB2/Zeo cells treated or not with PPMP. The violin plots were built with 1000 trajectories for each condition. The dotted lines indicate the median of the populations. *** indicate that the difference between the populations is significant with a *p* value below 0.0001 as determined by the Mann–Whitney U test. Right: histograms representing the percentage of tetraspanin molecules exhibiting Brownian, confined and mixed motion relative to the total number of trajectories. The error bars represent the standard deviation of at least three independent experiments. (**B**) Left: Distribution of the apparent diffusion coefficients calculated for all individual CD9 molecules at the plasma membrane of HB2/CD82 treated or not with PPMP. The violin plots were built with 1000 trajectories for each condition. The dotted lines indicate the median of the populations. «ns» indicate that the difference between the populations is not significant as determined by the Mann–Whitney U test. Right: Histograms representing the percentage of CD9 molecules exhibiting Brownian, confined and mixed motion relative to the total number of trajectories. Error bars represent the standard deviation of at least three independent experiments. (**C**) Ensemble labelling of CD81 and CD9 (Top panel), CD82 and CD81 (Bottom panel) in HB2/CD82 cells treated with PPMP. The scale bar represents 5 μm. Images on the right part correspond to zooms delineated by the red boxes and represent the merge of the two channels. The yellow pixels represent the colocalization between tetraspanins. The histograms represent the calculated Pearson’s correlation coefficients between the two signals in HB2/Zeo and HB2/CD82 cells using the «colocalizer studio» plugin of Icy software. Calculations were done on at least 10 cells and the error bars represent the standard deviation.

PPMP treatment of HB2/CD82 cells did not modify the lateral diffusion of CD9 at the plasma membrane (Figure 5B) but increased the diffusion of CD81. This effect of PPMP on CD81 dynamics did not require CD82 expression since PPMP had an even more pronounced effect on the diffusion of CD81 in HB2/Zeo cells (0.02 μm^2^/s versus 0.07 μm^2^/s upon PPMP treatment). By contrast, the increase in CD81 median diffusion in PPMP-treated cells appears to be a consequence of an increase in the proportion of Brownian trajectories since we observed no impact on the diffusion coefficient of CD81 molecules displaying Brownian motion after the treatment.

We also wanted to assess whether ganglioside depletion after the PPMP treatment affects cellular distribution of tetraspanins. TIRF microscopy analysis of ensemble labeling revealed that the distribution patterns of CD82, CD81 and CD9 are different in PPMP-treated HB2 cells (compare Figure 3 and Figure 5C). Specifically, the dot-like structures and patches observed in untreated cells are less prominent in the PPMP-treated cells with all three tetraspanins distributing more homogeneously at the basal membrane of HB2 cells. In addition, the accumulation of tetraspanins at the cell periphery was lost in these cells. upon ganglioside depletion. Interestingly, while the level of CD9 and CD81 colocalization in HB2/Zeo and HB2/CD82 cells was not affected upon PPMP treatment, co-localization between CD82 and CD81 in HB2/CD82 cells was diminished (indicated by the decrease of the PCC from 0.76 in untreated cells to 0.45 in PPMP-treated cells). These results suggest that there is specific and differential contribution of gangliosides in co-clustering of tetraspanins on the plasma membrane (Figure 5C). However, co-immunoprecipitation showed minor alterations if any of the association of CD82, CD9 and CD81 with one another, confirming that gangliosides are not essential for the interaction between these tetraspanins (Figure S5).

## 3. Discussion

It is now well established that tetraspanins form a dynamic network of protein–protein and protein–lipid interactions that likely defines their ability to diffuse in the plane of the plasma membrane. Previous studies have separately addressed the dynamics of CD9, CD81 and CD151 diffusion within the membrane of fibroblastic (CHO) [30], endothelial (HUVEC) [11] and epithelial cells (HeLa, PC3, HuH7) [13,14,30]. While clear differences in the behavior of these tetraspanins have been observed, it remained unknown whether this was due to intrinsic differences between tetraspanin proteins themselves or cell type specific differences in molecular composition of the plasma membrane. To address this question we compared the membrane dynamics of CD9 and CD81 (two closely related members of the tetraspanin family [36]), with that of CD82, a more distant tetraspanin (reviewed in [9]). We have also examined for the first time the contribution of gangliosides to the surface dynamics of tetraspanin proteins. Importantly, these experiments were performed in the context of one cellular model, which allowed us to draw a definitive conclusion on the specific contribution of gangliosides to the surface behavior of different tetraspanins. Our results demonstrated that all three tetraspanins display different membrane dynamics. Furthermore, we also showed that one tetraspanin can specifically affect the behavior of another one. Finally, we discovered that gangliosides could differentially contribute to the dynamics of tetraspanins on the cell surface.

Like many other membrane proteins, CD9, CD81 and CD82 display a combination of different diffusion modes, with a fraction of the molecules diffusing in a Brownian mode, while others are locally confined, either transiently or permanently. Interestingly, both apparent diffusion coefficients and motion types varied between the 3 tetraspanins in HB2 cells. Specifically, CD9 dynamics were higher than that of CD81 dynamics, which were themselves greater than that of CD82. As our previous work had already shown that CD9 was much more dynamic than CD81 at the plasma membrane [15], the higher dynamics of CD9 as compared to CD81 is likely to be due to different intrinsic properties between the two molecules. The lower diffusion coefficients calculated for CD81 and CD82, as compared with CD9, are due to a large extent to a higher fraction of confined molecules, which diffuse slowly. We have previously shown using a combination of single molecule tracking and ensemble labeling that CD9 molecules are confined in areas enriched in these molecules, which we referred to TEAs, suggesting that these TEAs correspond to the confinement of several molecules at the same place and at the same time [13]. By analogy, we can hypothesize that more CD82 molecules than CD81 molecules and more CD81 molecules than CD9 molecules are trapped in TEAs or that they are trapped for a longer time. In this regard, analysis of the distribution of these tetraspanins shows that there are more CD82- and CD81-enriched areas. The higher confinement of CD82 and CD81 may be due to a more favorable interaction with discrete proteins or lipids that serve as nucleation factors within the TEAs or stabilize them (see below). It cannot be explained by a differential expression of CD9 and CD81, which were comparable in HB2/Zeo and HB2/CD82 cells (Figure S2C). Importantly, the diffusion coefficients of molecules with Brownian behavior were also different between different tetraspanins, with CD9 Brownian molecules diffusing more than twice as fast as CD82 Brownian molecules (CD81 displayed an intermediate speed). A possible explanation is that CD82 (and to a lesser extent CD81) makes more contacts with membrane or submembrane components that restrict its diffusion. In this regard, a strong link between tetraspanins and the actin cytoskeleton has been previously reported [37]. Since the link between tetraspanins and the actin cytoskeleton is likely to be indirect, differential behavior of CD9, CD81 and CD82 may be explained by their specific preferences in “choosing” their molecular partners that connect them to the actin network. Consequently, membrane dynamics of tetraspanins may be determined by the size of the clusters formed with their specific partner proteins. Indeed, larger nanostructures diffuse slower according to the hydrodynamic model developed by Saffman and Delbrück that predicts a logarithmic dependence of the diffusion coefficient with the radius R of the diffusant [38]. CD82 may be embedded in larger membrane assemblies than CD81, which itself could be in larger assemblies than CD9. Alternatively, the lipid environment within or around the tetraspanin assemblies could explain the differential behavior of the tetraspanins. Indeed, tetraspanins were shown to directly interact [1,39,40] and the influence of cholesterol content into the membrane on the diffusion of transmembrane proteins has also been described for a few transmembrane proteins including CD9 and CD81 [15], Patched1, the receptor of the secreted Hedgehog ligand Sonic Hedgehog [41] or NrCAM, a cell adhesion molecule of the L1 family [42]. Moreover, our experiments involving ganglioside depletion strongly further support the idea that changes in the lipid composition of the plasma membrane may have a differential effect on the membrane dynamics of various tetraspanins (see below).

In line with the importance of the composition and the size of TEAs on tetraspanin membrane dynamics and partitioning, we demonstrate here that the expression of a particular tetraspanin protein could specifically influence the dynamics of another tetraspanin family member. Indeed, increased expression of CD82 specifically decreased the proportion of “confined” CD81 molecules at the basal membrane, thus leading to the increased number of CD81 molecules exhibiting Brownian motion. Given that the diffusion coefficient of Brownian CD81 molecules was not affected by CD82 expression, these results suggest that CD82 may be involved in displacement of CD81 from the TEAs. Importantly, the modulation of CD81 dynamics by CD82 expression was specific since the behavior of CD9 and α_3_β_1_ integrin was not affected. Our results also suggest that while CD82 does not seem to affect the co-localization between CD9 and CD81, it may modify cellular distribution of CD81 and CD9 by directing the proteins to the periphery of the cells. Similarly, several tetraspanins have been described to be enriched at the periphery of breast cancer cells [43] and we have shown that expression of TSPAN5 in U2OS cells led to an enrichment of its partner ADAM10 at the cell periphery [44]. While molecular pathways linking CD82 expression with redistribution of other tetraspanins require further investigation, it is tempting to speculate that the underlying mechanisms may involve other membrane tetraspanin partners. For example, we found that EWI proteins, which have been described as primary partners of CD81 and CD9, also interact with CD82 in HB2 cells as shown with immunoprecipitation experiments (Figure S5).

Gangliosides are key components of the plasma membrane in eukaryotic cells and have been associated with a large variety of cellular processes, especially in the formation and function of microdomains. We investigated the effects of ganglioside expression on the behaviors of the tetraspanins CD82, CD9 and CD81 by tuning the expression of these lipids with PPMP. PPMP treatment was associated with a strong increase in CD82 and CD81 dynamics but not in CD9 dynamics, showing that the effects of PPMP are not due to the general change in membrane properties. This increase is mainly due to a decrease in the number of confined CD82 and CD81 molecules, indicating that confinement of these molecules, possibly in TEA, is highly dependent on the presence of gangliosides. However, the intrinsic link between gangliosides and these tetraspanins may be different as the diffusion coefficient of Brownian CD82 molecules (but not that of Brownian CD81 molecules) was increased upon ganglioside depletion. These observations are consistent with our previous work showing that ganglioside depletion induced CD82 partitioning into the light fraction of the sucrose density gradient, indicating a change in membrane environment [35]. The co-localization experiments performed here further support the role of gangliosides in surface distribution of tetraspanins. Indeed, the size of tetraspanin assemblies observed in HB2/Zeo and HB2/CD82 cells were reduced in cells treated with PPMP and tetraspanins were more homogeneously distributed. Interestingly we discovered that ganglioside depletion only affected co-localization between CD9 and CD81, thus highlighting the role of gangliosides in the structural heterogeneity of tetraspanin clusters on the cell surface [45]. In addition, a specific increase in the diffusion coefficient of Brownian CD82 molecules was observed upon ganglioside depletion (no modifications in the Brownian diffusion coefficients were observed for other tetraspanins or for α3 integrin). Since CD82 has been proposed to directly interact with gangliosides GM2, GM3 and GD1a [46,47], this interaction could explain why only Brownian CD82 molecules are sensitive to ganglioside depletion. Importantly, despite these modifications of membrane compartmentalization, lowering the level of gangliosides yields only minor [35] or no change (this study) in the interaction of CD82 with CD9 and CD81. Thus, gangliosides are dispensable for these interactions and are probably more involved in the dynamic behavior of diffusing CD82 proteins (in agreement with the study of Aikihiro Kusumi’s group describing gangliosides as lipids that are very dynamic molecules moving in and out of membrane microdomains in an extremely dynamic manner [48]). It is therefore possible that transient interaction of gangliosides with CD82 could increase the Brownian diffusion coefficient of this tetraspanin.

In conclusion, we emphasized in this work that both lipid and protein compositions of the plasma membrane are involved in the dynamical architecture of the tetraspanin web. Importantly, each tetraspanin appears to have a specific signature in terms of dynamics, which is partly based on the ganglioside composition of the plasma membrane. These observations lay a solid foundation for further analysis focused on the role of tetraspanins in regulation of the membrane dynamics and, ultimately, functionalities of tetraspanin-based protein complexes.

## 4. Materials and Methods

### 4.1. Antibodies

Fab fragments or full length mAbs raised against CD81 (TS81), CD9 (SYB-1), and CD82 (TS82) were produced, purified and labeled with Atto647N or Cy3B as previously described [13,14]. The three antibodies recognize the extracellular loops of the corresponding tetraspanin [49,50].

### 4.2. Cell Culture and Treatments

HB2 mammary epithelial cells isolated from human breast milk were provided by Dr. Fedor Berditchevski and Dr. Elena Odintsova. Different cell lines were used: HB2/Zeo cells weakly expressing CD82 and HB2/CD82 cells overexpressing CD82. All these lines were cultured in a complete DMEM medium containing 10% heat inactivated fetal bovine serum (FBS), 1 mM pyruvate, 10 μg/mL hydrocortisone and 10 μg/mL insulin. Cells in culture were tested for mycoplasma contamination using MycoAlert Mycoplasma Detection kit from Lonza according to manufacturer instructions. 

For single molecule tracking experiments, around 10^5^ cells were plated 24 h before the experiment in 6-well culture dishes containing 25 mm diameter coverslips that had been previously cleaned using plasma etcher.

For PPMP treatment, cells were grown to a confluence around 50% and D-threo-1-phenyl-2-hexadecanoylamino-3-morpholino-1-propanol·HCl (PPMP) purchased from Matreya was added to reach a final concentration of 2 μM (this concentration was tuned in order to prevent cell death). After one-day treatment, the medium was removed and a fresh medium containing 5 μM of PPMP was added. After two days of culture, the medium was again exchanged for medium containing 10 μM and then routinely cultured as described above. Treatment efficiency was either assessed by flow cytometry using anti-GD1a antibody or by mass spectrometry.

### 4.3. SiRNA Experiments

Cells were transfected with Lipofectamine 2000 purchased from ThermoFisher. For CD82 expression knockdown, the following duplex was used: GCTGGGTCAGCTTCTACAAdTdT and TTGTAGAAGCTGACCCAGCdCdG. Briefly, cells were plated on 6-well dishes at 70–80% confluence the day prior to the transfection. For each well, 5 μL of Lipofectamine 2000 were added to 250 μL of Opti-MEM and the CD82 siRNA was added to a final concentration of 100 pM in 250 μL of Opti-MEM. The two samples were incubated at room temperature for 20 min and then mixed together. The complete medium of each well was then exchanged for 2 mL of Opti-MEM and the transfection mixture was added to each well. After 8 h, the Opti-MEM was removed and 2 mL of complete medium was added to each well. The following day, cells were detached and plated on 25 mm coverslips for further single molecule tracking experiments. After the experiments, the efficiency of CD82 siRNA was assessed by flow cytometry on cells used for the tracking. 

### 4.4. Single-Molecule Tracking

SMT experiments were carried out as previously described [13]. Briefly, cells plated on coverslips were incubated in red phenol-free DMEM at 37 °C for 10 min with Atto647N-labeled Fab fragments of mAbs raised against CD81 (TS81), CD9 (SYB-1) or CD82 (TS82) at concentrations in the range of 1 to 10 ng/mL. For single molecule experiments, ~ one probe per Fab is required. Ensemble labeling was performed with full antibody. Homemade objective-type TIRF setup allowing multicolor single-molecule imaging and equipped with a Plan Fluor 100×/1.45 NA objective (Zeiss, Le Pecq, France Brattleboro, VT) was used. All the experiments were performed with a 100 ms integration time. The localization of each fluorescence peak was determined with subpixel resolution by fitting a two-dimensional elliptical Gaussian function. The accuracy of the position measurement in living cells was estimated to be 50 nm by fitting a 2D Gaussian to the emission intensity distribution of an immobile single molecule conjugated with Atto647N.

Movies were analyzed using homemade software (named ‘PaTrack’) implemented in visual C++ ([29], freely available using the link). Trajectories were constructed using the individual diffraction limited signal of each molecule. The center of each fluorescence peak was determined with subpixel resolution by fitting a two-dimensional elliptical Gaussian function. The two-dimensional trajectories of single molecules were constructed frame per frame. Only trajectories containing at least 41 points were retained. Diffusion coefficient values were determined from a linear fit to the MSD (mean square displacement)-τ plots between the first and the fourth points (D1–4) according to the equation MSD(t) = 4Dt. The determination of the motion modes was performed using homemade algorithm based on a neural network that has been trained using synthetic trajectories to detect pure Brownian, confined and directed motion modes. Thanks to a sliding window, the trajectory is analyzed and the different modes can be confidently detected within a trajectory for segments larger than 10 frames. Once the motion mode is identified, the different segments are analyzed by plotting the MSD versus time lag. The MSD curve was linearly fitted (Brownian) or adjusted with a quadratic curve (4Dt + v2t2) (directed diffusion) or exponential curve L2/3(1–exp(–12Dt/L2) (confined diffusion), where L is the side of a square domain, the confinement diameter being related to L by Ø conf = (2/√L) [51]. The apparent diffusion coefficient values were determined from a linear fit between the first and fourth points (D1–4). The algorithm was tested with simulated trajectories displaying pure Brownian, confined or directed behavior or a combination of these 3 modes and successfully applied to a set of single molecule experiments previously recorded for tetraspanins diffusing into plasma membranes.

### 4.5. Flow Cytometry Experiments

Cells were grown in 25 cm^2^ flasks until reaching exponential growth. Then, cells were harvested using an enzyme-free cell dissociation reagent (purchased from Gibco) and centrifuged at 100× *g* for 5 min at 4 °C. The cell pellet was thoroughly suspended in cold PBS. The cells were then centrifuged again and the pellet was suspended in PBS/1% FBS buffer containing 2 μg/mL of mouse IgG raised against the protein of interest. After 30 min of incubation, cells were centrifuged at 100× *g* for 5 min. The pellet was then suspended in PBS/1% FBS. This washing step was repeated 3 times. The pellet was suspended in PBS/1% FBS containing 1 μg/mL of secondary antibodies raised against mouse IgG and coupled to Alexa568. As a control, one sample was incubated only with the secondary antibody. After one hour of incubation at 4 °C in the dark, the washing steps were repeated 3 times. After the last wash, the cell pellet was suspended in 500 μL of cold PBS with 2% PFA. After 10 min, cells were washed to remove PFA and were analyzed using a Bio-Rad flow cytometer. Post-acquisition analyses were performed using the software FlowJo.

### 4.6. Dual-Color Immunofluorescence Using TIRF Microscopy

One day prior to the experiment, 2 × 10^5^ cells were plated in 6-well culture plates containing 25 mm diameter coverslips that had been previously plasma-cleaned. Cells were then fixed with PBS buffer containing 4% PFA for 10 min. Cells were then washed 3 times with PBS. PBS/1% FBS buffer containing 5 μg/mL of antibodies raised against the proteins of interest and coupled to Cy3b or Atto647N was then added to the coverslips for 30 min. Cells were then washed 3 times with PBS/1% FBS. Coverslips were mounted on a glass slide using Prolong Diamond and incubated overnight at 4 °C. Cells were imaged using a TIRF microscope at 10 images/s. One hundred frames were acquired and then stacked. Pearson’s correlation coefficients were calculated using the colocalizer studio plugin of the software Icy.

### 4.7. Mass Spectrometry Analysis of Lipids

Total lipids were extracted overnight at 4 °C from the samples dissolved in 0.5 mL water with 10 volumes (5 mL) of CHCl_3_/CH_3_OH (1:1, *v*/*v*). The residual pellet obtained after centrifugation (1500× *g*, 5 min) was extracted twice with 2 mL of the same solvent. The three lipid extracts were pooled, dried under a stream of nitrogen, and solubilized in 3 mL CHCl_3_/CH_3_OH (1:1, *v*/*v*). Gangliosides were then separated from other lipids using phase partition by adding 1 mL water. After centrifugation, the upper aqueous phase was collected while the lower organic phase was reextracted twice with 2 mL CH_3_OH/water (1:1, *v*/*v*). The three upper phases containing gangliosides were combined, dried under a stream of nitrogen, and solubilized in 2 mL CH_3_OH/PBS 10 mM (1:1, *v*/*v*). This ganglisoside extract was desalted on a C18 silica gel column (Sep-Pak Vac 6 cc, 500 mg; Waters), washed with 7 mL CH3OH and pre-equilibrated with 7 mL CH_3_OH/PBS 10 mM (1:1, *v*/*v*) before injection. After washing with 10 mL water, purified gangliosides were eluted with 6 mL CH_3_OH and 4 mL CHCl_3_/CH_3_OH (2:1, *v*/*v*). Liquid chromatography (LC) was performed at 30 °C using a Dionex UltiMateTM 3000 LC system from ThermoScientific equipped with an autosampler. Separation of GM3, GM2, GD3, GD1a, GD1b, GT1b, and GQ1b standards was achieved under hydrophilic interaction liquid chromatography (HILIC) conditions using a silica Kinetex column (GM1 measurement was not available when we have performed the experiments). The mobile phase was composed of acetonitrile/water (90:10, *v*/*v*) containing 10 mM ammonium acetate and acetonitrile/water (50:50, *v*/*v*) containing 10 mM ammonium acetate. The solvent-gradient system was as follows: 0–1 min 100%, 4 min 79%, 9 min 78%, 14–18 min 50%, and 19–48 min 100%. The flow rate was 400 μL/min and the injection volume was 10 μL. Eluates from the HPLC system were then analyzed by mass spectrometry (LC-MS/MS).

The cholesterol levels were analyzed using a Cholesterol Quantitation Kit from Sigma following the manufacturer’s protocol.

### 4.8. Immunoprecipitations of Tetraspanins and Partners

Cells were grown in 75 cm^2^ flasks until reaching 80% confluence. Cells were then washed three times with PBS and incubated with 10 mL of biotin at 0.5 mg/mL for 45 min at 4 °C. The cells were then washed three times with PBS and lysed in a buffer containing 30 mM Tris pH 7.4, 150 mM NaCl, protease inhibitors and 1% of Brij97. After 30 min of incubation at 4 °C, the insoluble material was removed by centrifugation at 10,000× *g* for 15 min. The cell lysate was then incubated with inactivated goat serum and G-sepharose protein beads for 2 h. The beads were removed by centrifugation and the lysate was incubated with 1 μg of primary antibody and 10 μL of G-sepharose protein beads for 400 μL of lysate for 2 h at 4 °C on a rotating wheel. The beads were washed four times using the lysis buffer with 0.5% Brij97 and Laemmli buffer was added. Immunoprecipitated proteins were separated by SDS-PAGE under non-reducing conditions and transferred to a low fluorescence PVDF membrane. Immunoprecipitates were analyzed by Western blot using streptavidin coupled to Alexa680 and blots revealed using the Odyssey Infrared Imaging System (LI-COR Biosciences GmbH, Bad Homburg, Germany). The antibody TS82 was coupled to biotin and revealed using streptavidin coupled to Alexa680.

## Data Availability

Data is contained within the article or Supplementary Material.

## Supplementary Materials

The following are available online at https://www.mdpi.com/article/10.3390/ijms22168459/s1.
