# Supplementary material for: CD82 and Gangliosides Tune CD81 Membrane Behavior
Source: Int J Mol Sci. 2021 Aug 6;22(16):8459. doi: 10.3390/ijms22168459 (PMC8395132; doi:10.3390/ijms22168459)
Supplement: Supplementary file 1 [file ijms-22-08459-s001.zip › ijms-1287497-supplementary.pdf]

Supplementary materials

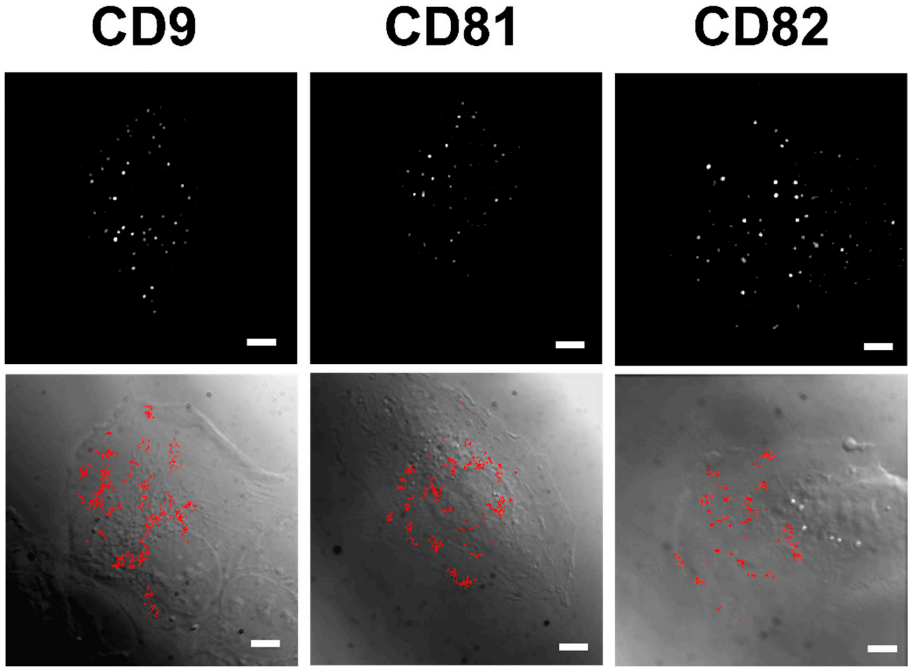

Figure S1. Representative experiments of single molecule tracking of tetraspanins CD9, CD81 and CD82 in HB2/CD82 cells. The top panel shows the first frame of the single molecule tracking movies. Each white dot is a single molecule of CD9, CD81 or CD82. The bottom panel shows the trajectories in red obtained from the corresponding single molecule movies superposed to the DIC images where cells can be observed. Scale bars, 5 μm.

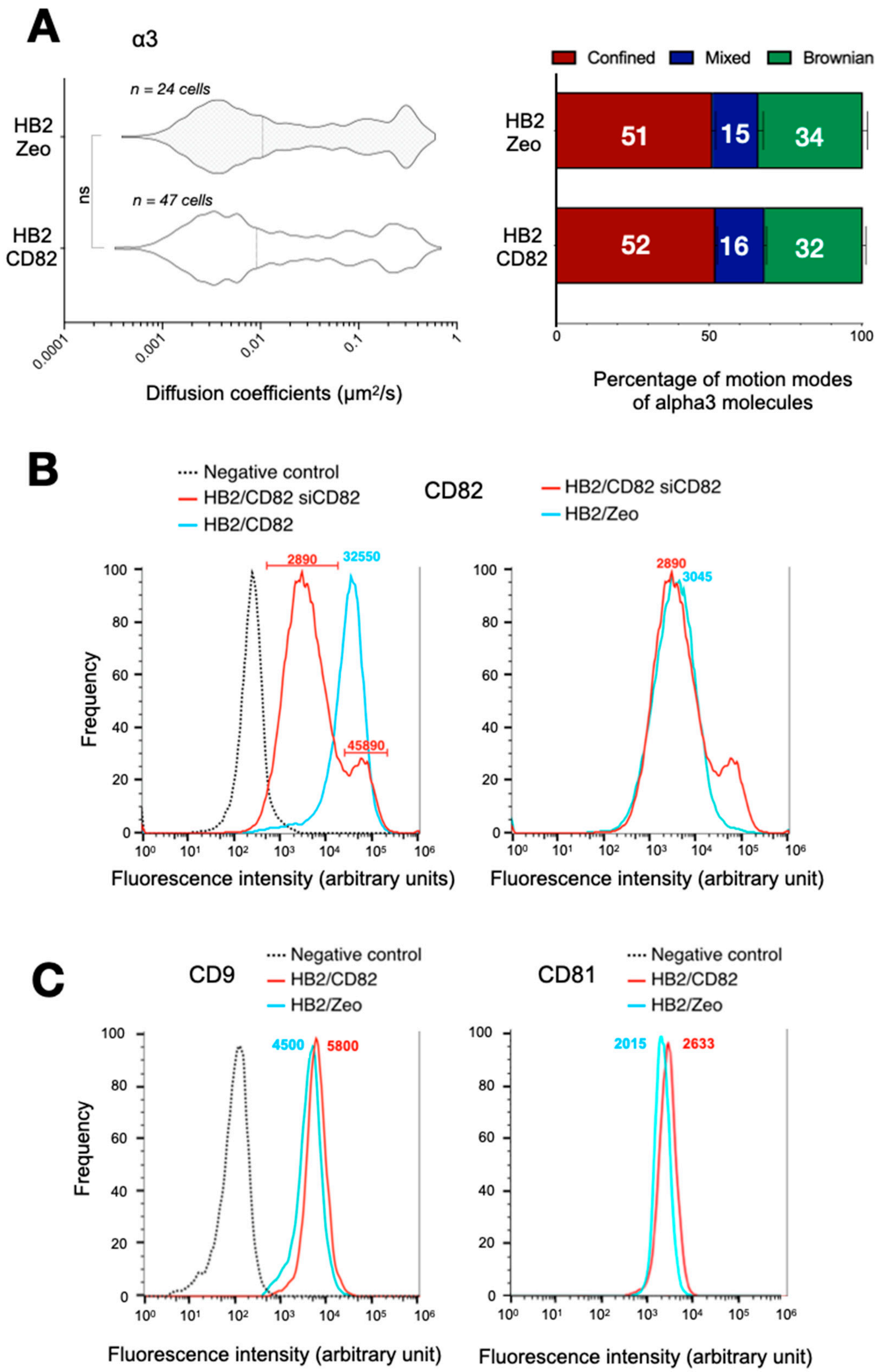

Figure 2. A) Left: Diffusion coefficients calculated for all individual α3 integrin molecules analyzed in HB2/CD82 and HB2/Zeo cells. The violin plots were built with 1000 trajectories for each condition. The dotted lines indicate the median of the populations. « ns » indicates that the difference between the populations is not significant as determined by the Mann–Whitney U test. Right: Histogram representing the percentage of α3 integrin molecules exhibiting Brownian, confined and mixed motion relative to the total number of trajectories. Error bars represent the standard deviation of at least three independent experiments. B) Downregulation of CD82 expression analyzed by flow

cytometry. Left: Comparison of CD82 surface expression between HB2/CD82 cells and HB2/CD82 cells transfected with siRNA targeting CD82. More than 80% of HB2/CD82 transfected with siRNA show a 10-fold decrease in CD82 surface expression. Right: Comparison of CD82 surface expression between HB2/Zeo cells and HB2/CD82 cells transfected with siRNA against CD82. HB2/Zeo cells expressed the same quantity of CD82 at the cell surface compared to HB2/CD82 cells transfected with siRNA. Cells were labeled with TS82 and then with a secondary antibody labeled with Alexa568 and directed against mouse IgG. The negative control represents HB2/CD82 cells incubated only with the secondary antibody. The values represent the median of each population. C) Surface expression of CD9 (left panel) and CD81 (right panel) in HB2/Zeo and HB2/CD82 cells analyzed by flow cytometry. Cells were labeled with Syb-1 or TS81 antibodies and then with a secondary antibody labeled with Alexa568 and directed against mouse IgG. The negative control represents cells incubated only with the secondary antibody. The values represent the median of each population.

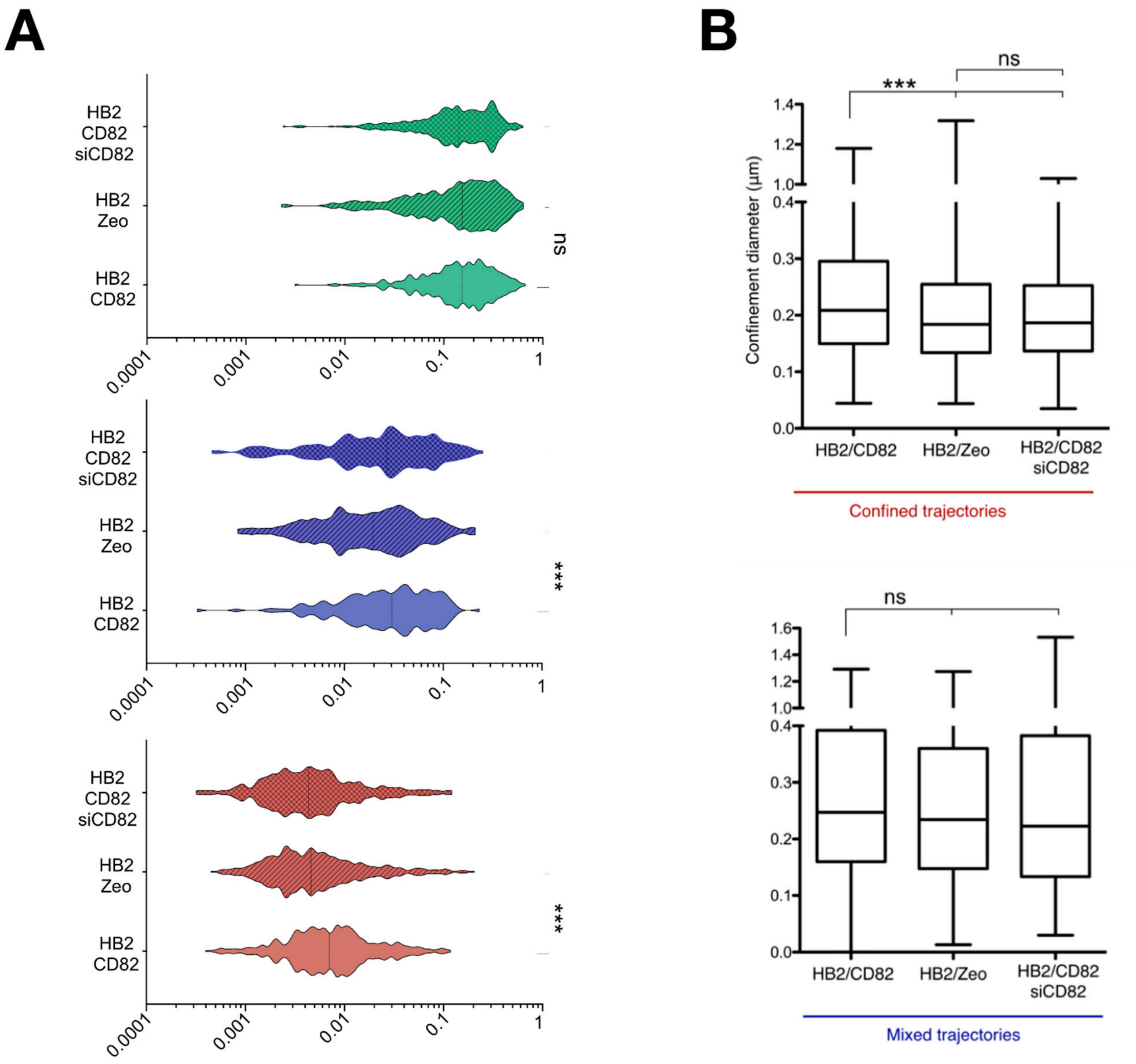

Figure S3. A) Distribution of the apparent diffusion coefficients calculated for all individual CD81 molecules displaying Brownian (green), confined (red) or mixed (blue) motions analyzed in HB2/CD82, HB2/Zeo cells or CD82-knockdown HB2/CD82 cells. The dotted lines indicate the median of the populations. Triple asterisks indicate that the difference between the populations is significant with a p value below 0.0001 as determined by the Mann-Whitney U test (« ns » for non-significant). B) Box-and-whisker representation of the confinement diameters of confined and mixed CD81 molecules in HB2 cells. The boxes represent 50% of the populations, the black lines are the medians of the populations. The whiskers represent the lower and upper quartiles. Triple asterisks indicate

that the difference between the populations is significant with a p value below 0.0001 as determined by the Mann-Whitney U test.

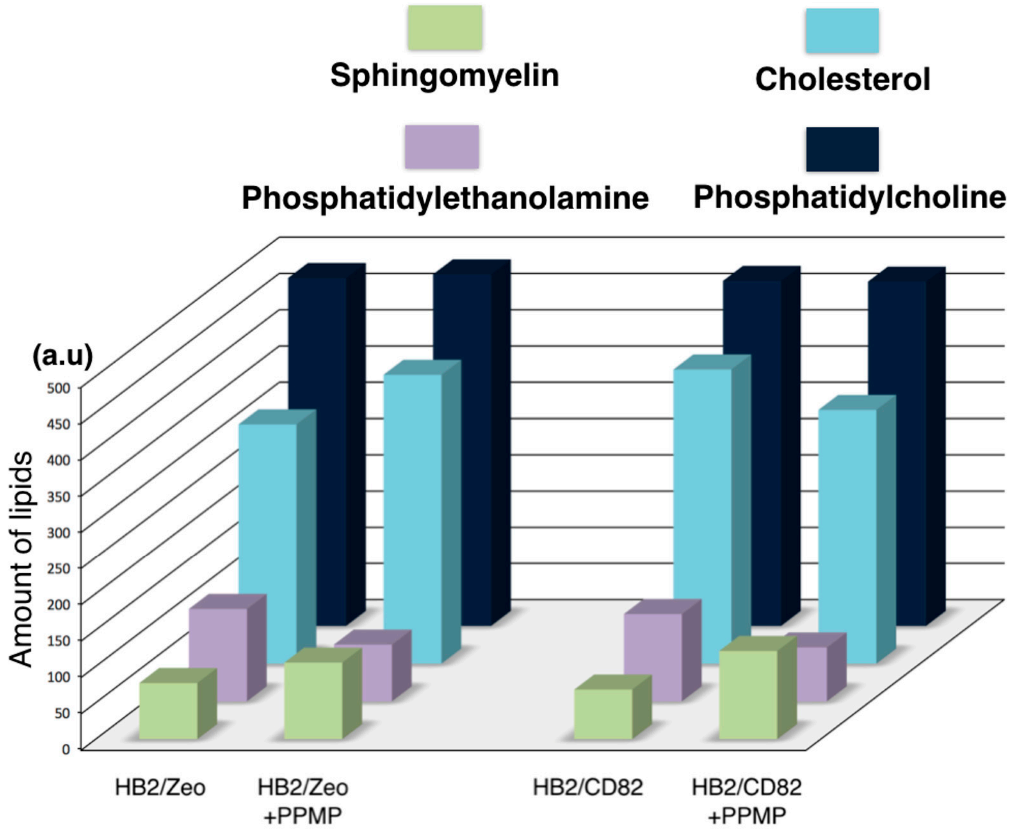

Figure S4. Mass spectrometry analysis of sphingomyelin, phosphatidylethanolamine and phosphatidylcholine levels in HB2/Zeo or HB2/CD82 treated or not with PPMP. The cholesterol levels were analyzed using a Cholesterol Quantitation Kit from Sigma.

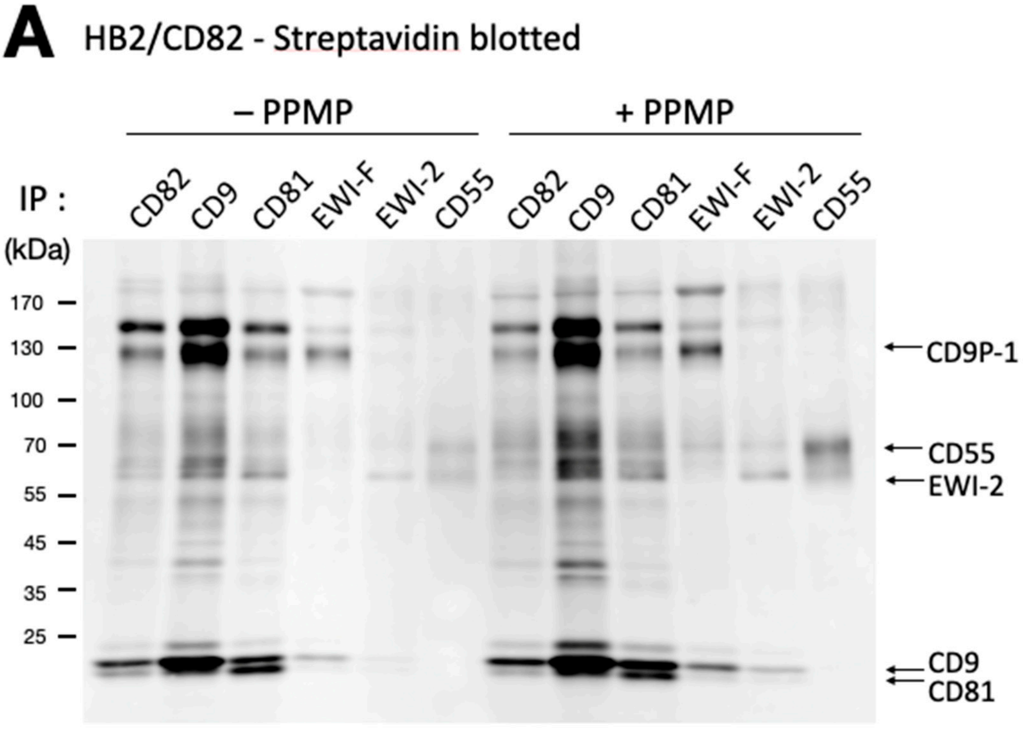

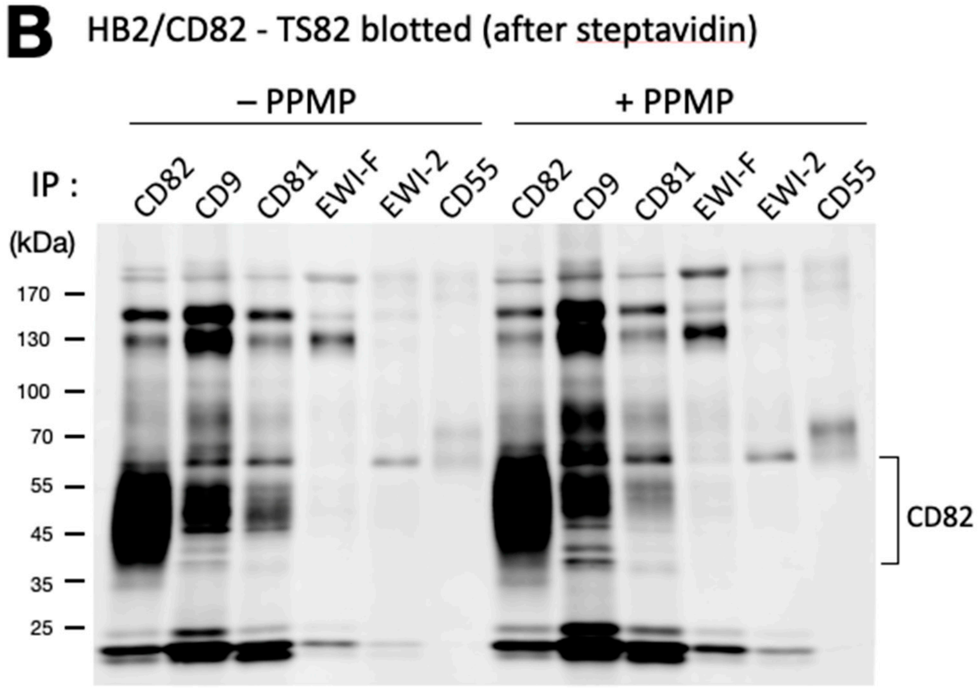

Figure S5. Immunoprecipitation**.** After biotin labeling of membrane proteins, HB2-CD82 cells, treated or not with PPMP, were lysed in the presence of Brij 97 before immunoprecipitation with the mAb as indicated at the top of each lane. The composition of the complexes was analyzed by Western blot using a combination of fluorescent streptavidin (panel A) or the biotin-labeled mAb TS82 to CD82 (panel B). All experiments were performed at least twice.

Supplemental movies

Movie 1: Representative single molecule movies of CD9 obtained in HB2/CD82 cells. Cells were labelled with fluorescent Fab fragments of antibodies at very low concentration and imaged in TIRF microscopy for one minute. The frame rate was 100 ms and the video shown are displayed in real-time. 29 µm x 21 µm images.

Movie 2: Representative single molecule movies of CD81 obtained in HB2/CD82 cells. Cells were labelled with fluorescent Fab fragments of antibodies at very low concentration and imaged in TIRF microscopy for one minute. The frame rate was 100 ms and the video shown are displayed in real-time. 29 µm x 21 µm images.

Movie 3: Representative single molecule movies of CD82 obtained in HB2/CD82 cells. Cells were labelled with fluorescent Fab fragments of antibodies at very low concentration and imaged in TIRF microscopy for one minute. The frame rate was 100 ms and the video shown are displayed in real-time. 29 µm x 21 µm images.
